# Variability and reproducibility of multi-echo *T*_2_ relaxometry: Insights from multi-site, multi-session and multi-subject MRI acquisitions

**DOI:** 10.3389/fradi.2022.930666

**Published:** 2022-07-28

**Authors:** Elda Fischi-Gomez, Gabriel Girard, Philipp J. Koch, Thomas Yu, Marco Pizzolato, Julia Brügger, Gian Franco Piredda, Tom Hilbert, Andéol G. Cadic-Melchior, Elena Beanato, Chang-Hyun Park, Takuya Morishita, Maximilian J. Wessel, Simona Schiavi, Alessandro Daducci, Tobias Kober, Erick J. Canales-Rodríguez, Friedhelm C. Hummel, Jean-Philippe Thiran

**Affiliations:** ^1^Signal Processing Laboratory 5 (LTS5), École Polytechnique Fédérale de Lausanne (EPFL), Lausanne, Switzerland; ^2^Translational Machine Learning Lab, Department of Radiology, Centre Hospitalier Universitaire Vaudois, University of Lausanne, Lausanne, Switzerland; ^3^CIBM Center for Biomedical Imaging, Lausanne, Switzerland; ^4^Department of Radiology, Centre Hospitalier Universitaire Vaudois, University of Lausanne, Lausanne, Switzerland; ^5^Defitech Chair for Clinical Neuroengineering, Neuro-X Institute (NIX) and Brain Mind Institute (BMI), École Polytechnique Fédérale de Lausanne (EPFL), Lausanne, Switzerland; ^6^Defitech Chair of Clinical Neuroengineering, Neuro-X Institute (NIX) and Brain Mind Institute (BMI), École Polytechnique Fédérale de Lausanne (EPFL Valais), Clinique Romande de Réadaptation, Sion, Switzerland; ^7^Department of Neurology, University of Lübeck, Lübeck, Germany; ^8^Center of Brain, Behavior and Metabolism (CBBM), University of Lübeck, Lübeck, Germany; ^9^Advanced Clinical Imaging Technology, Siemens Healthineers International AG, Lausanne, Switzerland; ^10^Department of Applied Mathematics and Computer Science, Technical University of Denmark, Kongens Lyngby, Denmark; ^11^Department of Neurology, University Hospital and Julius-Maximilians-University, Wuerzburg, Germany; ^12^Department of Neuroscience, Rehabilitation, Ophthalmology, Genetics, Maternal and Child Health (DINOGMI), University of Genoa, Genoa, Italy; ^13^Diffusion Imaging and Connectivity Estimation (DICE) Lab, Department of Computer Science, University of Verona, Verona, Italy; ^14^Clinical Neuroscience, University Hospital of Geneva (HUG), Geneva, Switzerland

**Keywords:** relaxometry, reproducibility, variability, MRI, multi-echo, quantitative MRI

## Abstract

Quantitative magnetic resonance imaging (qMRI) can increase the specificity and sensitivity of conventional weighted MRI to underlying pathology by comparing meaningful physical or chemical parameters, measured in physical units, with normative values acquired in a healthy population. This study focuses on multi-echo *T*_2_ relaxometry, a qMRI technique that probes the complex tissue microstructure by differentiating compartment-specific *T*_2_ relaxation times. However, estimation methods are still limited by their sensitivity to the underlying noise. Moreover, estimating the model's parameters is challenging because the resulting inverse problem is ill-posed, requiring advanced numerical regularization techniques. As a result, the estimates from distinct regularization strategies are different. In this work, we aimed to investigate the variability and reproducibility of different techniques for estimating the transverse relaxation time of the intra- and extra-cellular space ($T_{2}^{IE}$) in gray (GM) and white matter (WM) tissue in a clinical setting, using a multi-site, multi-session, and multi-run *T*_2_ relaxometry dataset. To this end, we evaluated three different techniques for estimating the *T*_2_ spectra (two regularized non-negative least squares methods and a machine learning approach). Two independent analyses were performed to study the effect of using raw and denoised data. For both the GM and WM regions, and the raw and denoised data, our results suggest that the principal source of variance is the inter-subject variability, showing a higher coefficient of variation (CoV) than those estimated for the inter-site, inter-session, and inter-run, respectively. For all reconstruction methods studied, the CoV ranged between 0.32 and 1.64%. Interestingly, the inter-session variability was close to the inter-scanner variability with no statistical differences, suggesting that $T_{2}^{IE}$ is a robust parameter that could be employed in multi-site neuroimaging studies. Furthermore, the three tested methods showed consistent results and similar intra-class correlation (ICC), with values superior to 0.7 for most regions. Results from raw data were slightly more reproducible than those from denoised data. The regularized non-negative least squares method based on the L-curve technique produced the best results, with ICC values ranging from 0.72 to 0.92.

## 1. Introduction

Quantitative magnetic resonance imaging (qMRI) has the potential to increase the specificity and sensitivity of conventional weighted MRI to underlying pathology. This increased sensitivity and specificity has stimulated the use of qMRI methods as potential biomarkers for microstructural integrity of the brain. Pathological processes such as demyelination, edema, iron accumulation, and tissue loss lead to variable and complex changes in tissue microstructure, inducing in turn changes in relaxation times. Therefore, these microstructural features can be inferred by *in-vivo* qMRI at millimeter resolution thanks to biophysical models. The spin-spin transverse relaxation rate *T*_2_ is one of the fundamental tissue-specific MRI contrast sources. In complex tissue, the microstructure can be seen as a combination of different pools of water with different chemical environments (called compartments) which each have their own characteristic *T*_2_. Hence, for complex tissues, multi-component *T*_2_ relaxometry allows probing the tissue microstructure by differentiating compartment-specific *T*_2_ relaxation times (1). Recent advances in multi-component *T*_2_ relaxometry acquisition (2, 3) and reconstruction methods (4) have boosted the use of this technique for the assessment of white matter (WM) integrity in general, but most notably for the determination of the myelin water content (5–11), and the *T*_2_ of the intra- and extra-cellular spaces ($T_{2}^{IE}$) (12).

As $T_{2}^{IE}$ is usually estimated from the *T*_2_ distribution by taking the (geometric) mean in the interval from 40 to 200 ms (at 3T), it is not affected by partial volume contamination from free water (*T*_2_>200 ms) or myelin water compartments (*T*_2_ <40 ms). This is the main advantage of using $T_{2}^{IE}$ over the mean intra-voxel *T*_2_ estimated from other qMRI techniques. Interestingly, a recent study revealed a strong correlation between $T_{2}^{IE}$ and age extending through the whole gray matter (GM), suggesting that this metric is sensitive to microstructural and macro-molecular content changes (13). However, multi-compartment *T*_2_ estimation methods are still sensitive to the underlying noise and the employed regularization technique for solving the resulting inverse problem (14, 15). This limitation is especially relevant for clinically achievable signal-to-noise ratios (SNR), which may affect the reproducibility and stability of the derived scalar maps. Several studies have already focused on the reproducibility and stability of myelin water fraction (MWF) estimates (16–20). However, the variability linked to the intra- and extra-cellular *T*_2_ component has been less studied, particularly in GM regions.

Other than the clinically achievable SNR, variability in qMRI data can be due to different factors such as hardware differences (scanner manufacturer, field strength, etc.), different MR reconstruction methods and acquisition parameters, among others. Even when the same hardware and sequence parameters are employed, high inter- and intra-scanner variability due to local and/or temporal scanner characteristics can occur. Characterizing this variability is challenging, mainly due to the lack of available data specifically designed to perform this task. In Cai et al. (21), authors tackle this issue with diffusion-weighted MRI data (DWI) using an *ad-hoc* data set (the MASiVar data set). The multi-site, multi-scanner, and multi-subject dataset proposed in that work was used to simultaneously characterize four commonly used diffusion processing techniques [diffusion tensor signal representation, multi-compartment neurite orientation dispersion and density imaging NODDI, microstructure model (22), white-matter bundle segmentation (23), and graph-based connectomics representations (24)].

In this study, we investigated the $T_{2}^{IE}$ variability and reproducibility in a clinical setting, in both GM and WM, using a multi-site, multi-session and multi-run dataset consisting of 20 healthy adults scanned in two different scanning sites, two separate sessions, and two runs (repetitions) per session and per site, resulting in a total of 160 scans (eight per subject). This unique dataset allows for quantifying the variability of the estimated multi-compartment *T*_2_ spectra, with respect to the subject, the acquisition site, session and run. Moreover, we aimed at evaluating the impact of using different techniques for estimating the *T*_2_ spectra, as well as the effect of the denoising of the data. In particular, three different non-parametric techniques were tested, including two types of regularized non-negative least squares (NNLS) methods based on the Chi-square (5) and L-curve (14) regularization criteria and a recently proposed Model-Informed Machine Learning approach using neural networks trained with synthetic data (25) on the original (raw) data and the same data after denoising.

## 2. Methods

### 2.1. Human brain MRI data

#### 2.1.1. Population

Twenty healthy subjects (nine female, mean age = 27, standard deviation = ± 3 years, age range = [24–33 years old]) were enrolled in the study. All subjects were right-handed and had no history of psychiatric diseases or any contraindications for performing MRI. Written informed consent was obtained from each participant following the Declaration of Helsinki. The ethics committee of the Canton of Vaud approved the study (Switzerland, project number: 2018-01355).

#### 2.1.2. MR acquisition

The MRI data were collected using a high-resolution 3D multi-echo gradient and spin-echo (GRASE) prototype sequence accelerated through CAIPIRINHA (3) with the following parameters: matrix-size = 144 ×126 ×134; voxel-size = 1.6 ×1.6 ×1.6 mm^3^; minimum echo time (TE) = 10.68 ms; Number-of-echoes = 32; Δ(TE) = 10.68 ms; repetition time (TR) = 1s; prescribed flip angle (FA) = 180°; number-of-slices = 84; acceleration factor = 3 x 2^(1)^; number of averages = 1; acquisition time = 10:30 min. T1-weighted images were also acquired for anatomical localization and definition of regions of interest (ROIs) using a 3D magnetization-prepared rapid gradient-echo (MP-RAGE) sequence with the following parameters: TR = 2,300 ms; Inversion Time (TI) = 7.1 ms; TE = 2.96 ms; FA = 9°; number-of-slices = 192; voxel-size = 1 ×1 ×1 mm^3^, field of view = 256 ×256 mm^2^.

#### 2.1.3. MRI scanning design

**Figure 1:**
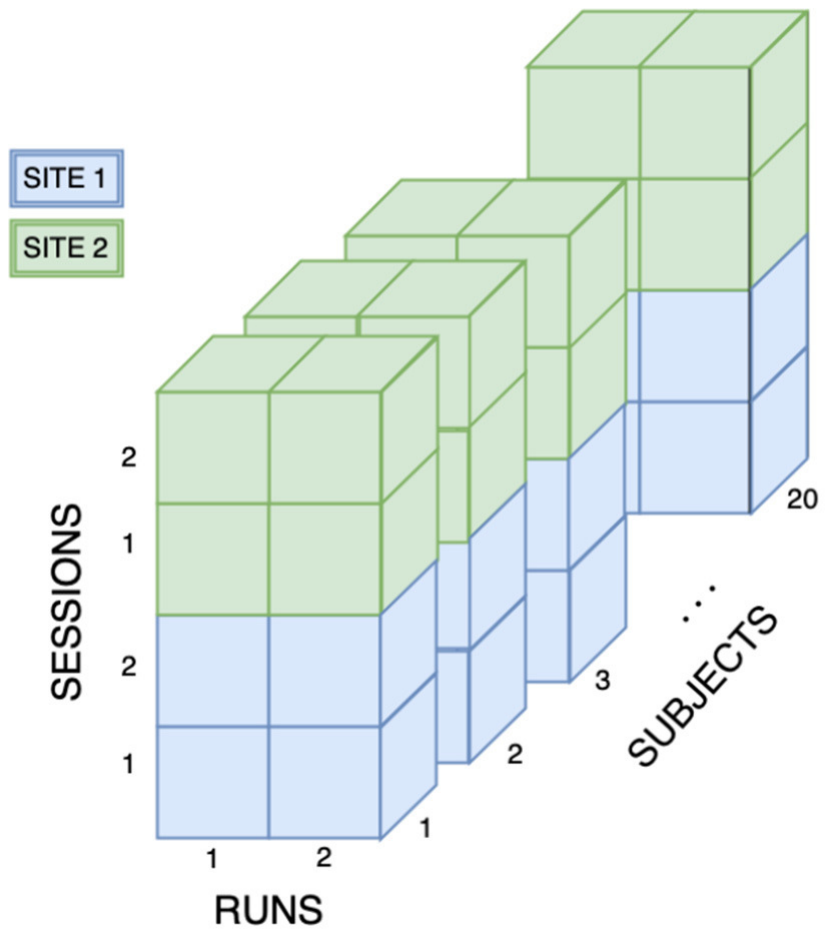
Overview of the data set. The cohort consists of 20 healthy subjects. All subjects were scanned on Siemens MAGNETOM Prisma (Siemens Healthcare, Erlangen, Germany) located at two different sites. Each subject underwent two sessions on each scanner and had two scans acquired per session, for a total of 160 scans.

Each subject was scanned in two MRI scanners (MAGNETOM Prisma, Siemens Healthcare, Erlangen, Germany) located at the Geneva University Hospital and Sion Hospital, Switzerland (sites) at two different points in time (sessions). At each session, each subject was scanned twice in two separate runs. Between the runs, subjects exited the scanner and were then repositioned, followed by a new shimming. Eight scans were obtained per subject, for a total of N = 160 scans. Figure 1 shows a schematic representation of the MRI scanning design. The mean elapsed time between intra-site and inter-site repetitions was 16 days (± 10 days) and 29 days (± 17 days), respectively.

### 2.2. MRI spatial processing

The T1-weighted image was normalized to the MNI standard space by registering them to the MNI152 template image using FSL FLIRT (26). Tissue partial volume estimates were obtained from the T1-weighted image using the FSL BET (27) and FSL FAST (28) methods. The definition of the different regions of interest, in both WM and GM, was performed using the Destrieux atlas available in FreeSurfer (29). Seventy-four regions per hemisphere were obtained for each tissue type. They were further grouped into five main cerebral lobes: parietal, occipital, temporal, the prefrontal part of the frontal lobe and its medial part.

### 2.3. *T*_2_ estimation

Two different scenarios were tested. In the first approach, the *T*_2_ estimation was performed over the raw data, without any prior denoising (raw data). For the second approach, the multi-echo *T*_2_ MRI data were filtered using a 3D total variation algorithm before fitting, using the *denoise-tv-chambolle* function in the *scikit-image* python toolbox (30) (denoised data). Spatial filtering is effective for decreasing the variability of the estimated maps [see (31–33)]. The noise standard deviation σ for each 3D volume was estimated by employing a robust wavelet-based estimator (34), and each volume was then denoised with a weight of 2σ as described in Canales-Rodríguez et al. (15). In both cases, the *T*_2_ spectra were estimated by using three different methods; two regularized non-negative least squares (NNLS) algorithms (*X*^2^−*I*, L-curve−*I*) and the Model-Informed Machine Learning (MIML) approach. More details are provided in the next two subsections.

#### 2.3.1. Regularized NNLS

The *T*_2_ spectra were computed in two steps. The refocusing flip angle (FA) value for each voxel was determined first, and then the intra-voxel *T*_2_ spectrum was computed by using the dictionary generated for the estimated FA. Estimating the optimal FA involves various steps. First, different matrices were generated using the Extended Phase Graph (EPG) model (35), each one corresponding to a fixed refocusing FA value selected from a discrete set of 15 equally spaced values between 90 and 180°. A fixed *T*_2_ range from 10 to 2,000 ms (36) with *N* = 60 *T*_2_ logarithmically spaced points was employed. Subsequently, we created a smoothed copy of the acquired multi-echo T2 data by using a Gaussian kernel (i.e., FWMH of 4.8 mm) as suggested in Drenthen et al. (20). Afterwards, the non-regularized NNLS algorithm was used to fit the smoothed data for each matrix, and the resulting mean square errors were interpolated using cubic B-splines (35). Next, the optimal FA was selected at the global minima of the resulting interpolated curve. Finally, a new matrix was generated using the estimated FA, which was used then for estimating the *T*_2_ spectrum (35). Note that the *T*_2_ spectrum and derived maps were computed from the TV-denoised data and not from the Gaussian-filtered copy, which was only employed to get a smooth FA map. To accelerate the estimation, instead of generating a new matrix for each estimated FA, we loaded the optimal kernel from a set of precomputed matrices which were created on a high-resolution grid of FA values from 90 to 180° with a step-size of 0.33°. The optimal kernel was selected by identifying the FA grid point closest to the interpolated FA.

As the estimated *T*_2_ distribution may depend on the chosen technique for determining the optimal regularization parameter, two approaches were implemented, a regularized NNLS method based on the Chi-square residual fitting criterion (5), and another based on the L-curve technique, as implemented in the multi-component *T*_2_ reconstruction toolbox (14): https://github.com/ejcanalesr/multicomponent-T2-toolbox. Both methods, named *X*^2^−*I* and L-curve−*I*, used an identity regularization matrix to promote smooth *T*_2_ distributions.

#### 2.3.2. Model-informed machine learning

The *T*_2_ distributions were obtained by using the MIML approach described in Yu et al. (25). It is based on a multilayer perceptron (MLP) which is trained to learn a map directly from the noisy multi-echo *T*_2_ signals to the corresponding *T*_2_ distributions. The MLP network was composed of 6 hidden layers with 256 neurons per layer and an output layer with 60 units, corresponding to the same resolution we used to solve the regularized NNLS problem described in the previous section. The hidden layers used a *ReLu* function as the activation function, while the output layer used a SoftMax activation function. The input to the network is a vector with 32 elements corresponding to the 32 echos of the acquisition sequence. The loss function is composed of two penalty terms, a squared L2 norm term and the Wasserstein distance on probability distributions.

This technique was implemented using TensorFlow 2.0 (37) on Python 3.6 (38) with an *Nvidia GTX 2070* GPU. The MLP network was trained using a total of 1,120,000 signal/distribution pairs. The optimization was carried out by using the Adam optimizer (39) with a learning rate of 5*e*^−4^, a batch size of 2,000, and 30 epochs. For more details, see Yu et al. (25). The trained MLP network was used to predict the *T*_2_ spectra from the measured multi-echo *T*_2_ data. The trained model and code are available at the following website: https://github.com/thomas-yu-epfl/Model_Informed_Machine_Learning.

### 2.4. Statistical analysis and evaluation metrics

In order to investigate all possible sources of variability in the (semi)-quantitative $T_{2}^{IE}$ maps in a clinical setting, four different effects were studied for both WM and GM. They included inter-run, inter-session, inter-scanner (i.e. inter-site) and inter-subject effects. The N = 160 scans were grouped in different subsets: (i) inter-run variability was computed using scans acquired within the same session of the same subject on the same scanner; (ii) for the inter-session variability, we used the scans acquired between different sessions of the same subject on the same scanner; (iii) inter-scanner variability included scans acquired between different sessions of the same subject on different scanners and (iv) finally, inter-subject was assessed averaging the scans of different subjects acquired in different sessions on the same scanner.

For each of the four effects studied, the variability was evaluated by computing variability within each of the aforementioned groups and then visualizing the distribution across groups on the inter-run, inter-session, inter-scanner and inter-subject levels. The variability was computed by means of the coefficient of variation (CoV), defined for each group as the standard deviation (SD) of the scalar metrics in each group, divided by the mean of the group, times 100. Intuitively, CoV is computed as the proportion of the average scalar measurement attributable to variability. As such, higher CoV indicates higher variability. The inter-run, inter-session, inter-scanner and inter-subject variability was independently computed on the $T_{2}^{IE}$ maps obtained for each of the reconstruction methods used (*X*^2^−*I*, L-curve−*I*, and MIML). For all reconstruction methods, the variability due to session, site and subject effects were compared pairwise using a Wilcoxon rank-sum test with Bonferroni correction.

In addition, the reproducibility of each reconstruction method (defined as the agreement of multiple assessments of the same subjects) was computed *via* the Intraclass Correlation Coefficient (ICC). While ICC is generally used to determine if subjects can be rated reliably by different raters, it can also be used for test-retest (repeated measures of the same subject).

The definition of the intraclass correlation is mainly based on analysis of variance or random effects models. While several ICC estimators have been proposed, most of the estimators can be defined in terms of the random effects model


(1) $$\begin{array}{l} Y_{ij}=\mu +\alpha_{j}+\varepsilon_{ij}, \end{array}$$

where *Y*_*ij*_ is the *i*^*th*^ observation in the *j*^*th*^ group. The terms μ, α_*j*_ and ϵ_*ij*_ are the unobserved overall mean, the unobserved random effect shared by all values in group *j* and the unobserved noise, respectively. Using a random effect framework, the population ICC can be defined as


(2) $$\begin{array}{l} \frac{\sigma_{\alpha }^{2}}{\sigma_{\alpha }^{2}+\sigma_{\varepsilon }^{2}}. \end{array}$$

The selection of the ICC statistics needs to be chosen carefully, as different statistics can produce different results for the same data. In this work, and following Koo and Li (40), we focus on the ICC for scan-rescan reliability study using a “two-way” random effects (i.e., considering both subjects and "raters" as random effects) a single repeated measurement model with "consistency" as the agreement term. ICC values range between 0 and 1, with a value of ICC close to 1 indicating high similarity between rater's scores (or repeated measurements) and ICC close to 0 showing low similarity of the values. According to Koo and Li (40), an ICC of less than 0.50 indicates poor reliability, while an ICC ranging from [0.5, 0.75] indicates a moderate one. Good reliability is indicated by an ICC value of 0.75 and higher, with excellent reliability defined as an ICC greater than 0.9.

For each reconstruction method, the ICC was computed by arranging the MRI scans in a matrix where rows represented subjects, and the columns represented each one of the repeated measurements. In order to assess the reproducibility of the MR measurements in specific brain regions and determine whether one region is more reproducible than others, several matrices were computed, where each cell of the matrix stored the mean $T_{2}^{IE}$ value within the ROI for each scan and reconstruction method. The ROIs under study comprised the whole WM and GM tissues as well as the prefrontal, frontal, parietal, temporal and occipital in WM and GM, independently. The ICC was computed in R Studio (Version 1.2.5042) using the *icc* function from the *irr* package https://cran.r-project.org/web/packages/irr/irr.pdf. Only the *single raters absolute* ICC was used. The confidence interval was set to 0.095.

## 3. Results

### 3.1. Inter-run, inter-session, inter-scanner and inter-subject variability

**Figure 2:**
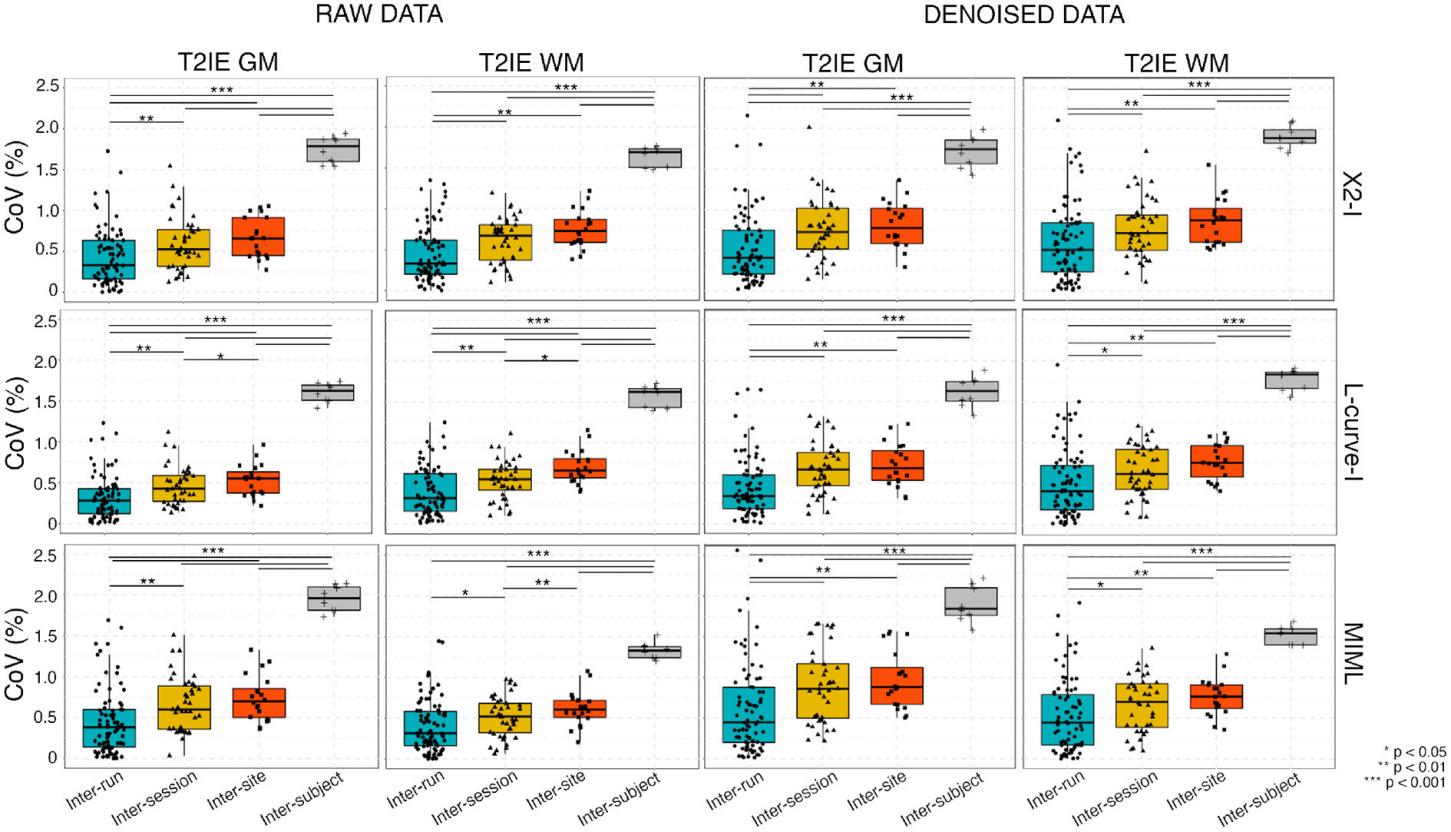
Variability of $T_{2}^{IE}$ for all reconstructions for both GM and WM for the raw data (without denoising, columns 1 and 2) and denoised data (columns 3 and 4): Coefficient of variation (CoV) across inter-run (scans acquired within the same session of the same subject on the same scanner), inter-session (scans acquired between different sessions of the same subject on the same scanner), inter-site (scans acquired between different sessions of the same subject on different sites) and inter-subject (average of the scans of different subjects in different sessions on the same scanner) groups. Increased variability is seen with session, scanner and subject effects, for both the raw data and the denoised data. Statistical differences between effects were assessed using a Wilcoxon rank-sum test with Bonferroni correction.

The inter-run, inter-session, inter-scanner and inter-subject variability for each reconstruction method in WM and GM tissue independently for both the raw data and the denoised data is shown in Figure 2 (from top to bottom, *X*^2^−*I*, L-curve−*I*, and MIML methods).

For all reconstruction methods under study in both the raw and the denoised cases, the mean CoV ranged between 0.32 and 1.64%. Overall, results from the raw and denoised data were similar. The highest $T_{2}^{IE}$ variability was found for the inter-subject effect, which was higher than the CoV induced by different sessions and sites for all the reconstruction methods in both GM and WM. This increased variability speaks in favor of the specificity of this metric for characterizing brain tissues. Moreover, our results demonstrate that, as expected, the variability consistently increases when changing session and site, with inter-run being the least variable followed by inter-session, and then inter-scanner and inter-subject for all the estimation techniques. Interestingly, the inter-session variability was close to the inter-scanner variability in both WM and GM. The pairwise comparison of the 4 effects show statistical significant pairwise differences for all effects besides between inter-session and inter-site effects (see Figure 2).

### 3.2. Variability in brain lobes

**Figure 3:**
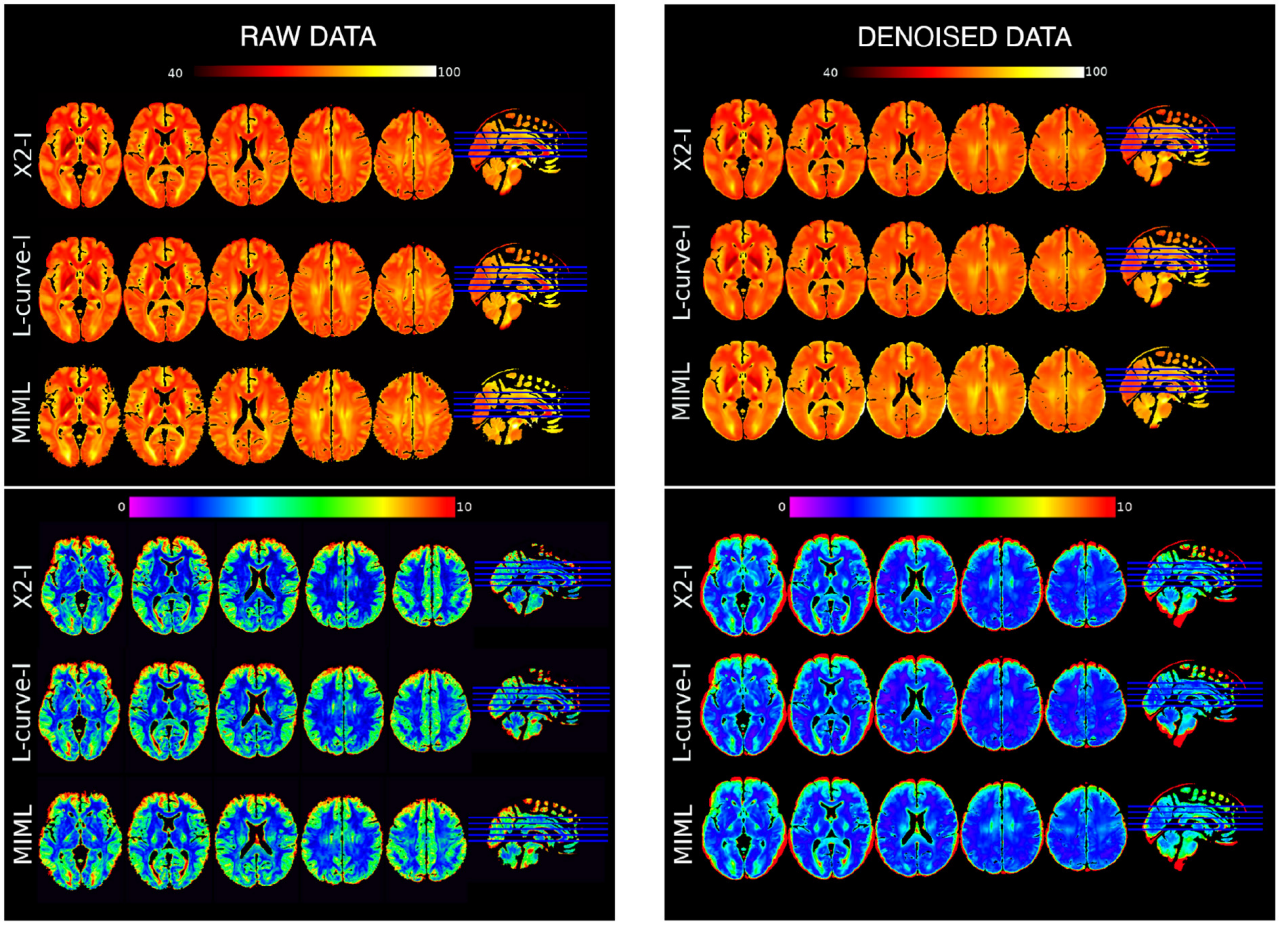
Whole-brain voxelwise *T*2^*IE*^ mean (upper panels, hot colormap) and standard deviation (lower panels, cold colormap) maps for the three reconstruction methods: *X*^2^−*I*, L-curve−*I*, and MIML. Left column, raw data. Right column, denoised data.

The voxelwise regional variability for each reconstruction method is represented in Figure 3, which shows heat maps of $T_{2}^{IE}$ mean (first panel) and $T_{2}^{IE}$ standard deviation (second panel) across the whole dataset. While the raw and denoised datasets appeared qualitatively similar, more anatomical details can be seen in the mean maps estimated from raw data. Overall, the mean $T_{2}^{IE}$ was higher in GM, and some WM regions, including the corticospinal tract and the optic radiations.

Table 1 show the mean and standard deviation values of the *T*2^*IE*^ in the different brain regions for both WM and GM for all reconstruction methods under analysis. All three reconstruction methods showed consistent results, although the MIML method displayed a higher mean $T_{2}^{IE}$ values. The values obtained with the NNLS methods were almost identical, with only slight differences mostly related to the smoothness of the solution. On the other hand, the variability (measured by the standard deviation) is higher in frontal regions for all the reconstruction techniques.

**Table 1:** $T_{2}^{IE}$ mean (standard deviation) in WM and GM regions for all reconstruction methods under analysis.

	**RAW DATA**			**DENOISED DATA**		
**ROI**	**L-CURVE-I**	**X2-I**	**MIML**	**L-CURVE-I**	**X2-I**	**MIML**
GM (whole brain)	68.09087 (1.074768)	67.31724 (1.154775)	69.34736 (1.33019)	69.19943 (1.105125)	68.55026 (1.1633760)	71.31032 (1.340239)
Prefrontal GM	72.54601 (7.8579)	72.23892 (8.124845)	76.43832 (6.546387)	72.88112 (7.50065)	72.43613 (7.8527090)	76.21879 (6.583347)
Frontal GM	71.46121 (4.971109)	71.47943 (4.942458)	76.81224 (4.137405)	73.01721 (4.285368)	72.49305 (4.433513)	76.45853 (3.986504)
Parietal GM	72.50995 (3.400083)	72.38689 (3.376828)	76.1375 (2.455073)	73.00612 (2.770335)	72.77265 (2.849852)	76.00545 (2.688274)
Temporal GM	73.57243 (3.604324)	73.39499 (3.891249)	79.01297 (2.923089)	73.91503 (3.68813)	73.30519 (4.158284)	79.24136 (3.283316)
Occipital GM	70.42572 (1.982738)	69.51394 (2.066751)	72.34303 (2.929528)	70.01067 (2.027722)	69.21856 (2.129861)	72.06157 (3.041027)
WM (whole brain)	71.68393 (1.123686)	71.5595 (1.165196)	76.91091 (1.005409)	72.69097 (1.272473)	72.28397 (1.350981)	76.87399 (1.147712)
Prefrontal WM	65.8683 (2.772873)	64.89867 (2.933406)	67.18295 (3.023913)	67.31196 (2.875424)	66.51591 (3.110145)	69.46465 (3.057553)
Frontal WM	68.67319 (2.961073)	67.7981 (3.022375)	69.8067 (3.290552)	70.14292 (2.638769)	69.12846 (2.707075)	71.52526 (3.074072)
Parietal WM	68.75922 (2.078946)	67.91948 (2.005582)	69.85067 (2.294526)	69.71293 (1.960121)	69.11882 (1.95721)	72.01084 (2.35551)
Temporal WM	68.1875 (2.561752)	67.58988 (2.742603)	70.06063 (2.503632)	69.64208 (2.399448)	69.23569 (2.538462)	72.58441 (2.308901)
Occipital WM	69.34293 (2.868029)	68.95491 (3.168218)	71.22032 (3.485063)	69.38705 (2.224087)	69.26719 (2.5307)	72.14998 (2.709813)

### 3.3. Reproducibility of each reconstruction method

**Figure 4:**
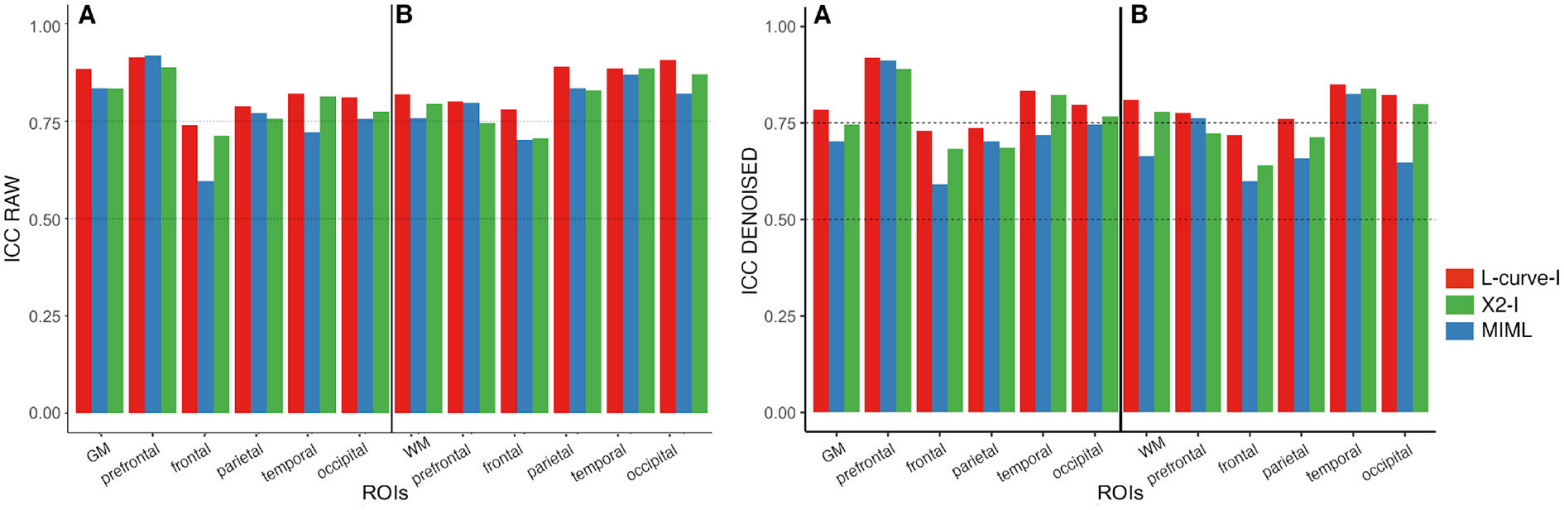
Regional ICC of Mean $T_{2}^{IE}$ for all three reconstruction methods for raw (left plot) and denoised data (right plot). Results for GM and WM are displayed in sub-panels (A,B), respectively. In each plot, the following anatomical regions are shown, from left to right: whole-brain (GM/WM), prefrontal, frontal, parietal and temporal regions. Color bars indicate different reconstruction methods: Red: L-curve−*I*, Green: *X*^2^−*I*, Blue: MIML.

We investigated the reproducibility of each reconstruction method by means of the ICC. The result of the voxel-wise ICC in the whole brain GM and WM, as well as different brain regions, for both the raw and denoised data is shown in Figure 4.

For most GM and WM regions, the ICC of $T_{2}^{IE}$ was higher when the *T*_2_ spectra were estimated using regularized NNLS methods (a few exceptions are the prefrontal in GM and WM, and the parietal in GM). When comparing the two NNLS methods under study, the one based on the L-curve−*I* criterion performed better. It produced the highest ICC scores for almost all the GM and WM regions, with values ranging from 0.72 to 0.92. Concerning the reproducibility of the reconstruction methods in specific brain regions, all three methods behaved similarly. Overall, the ICC in the whole GM and WM was moderate to good for the NNLS methods (ICC > 0.72 for L-curve−*I*, and > 0.70 for *X*^2^−*I*), while the MIML method achieved an acceptable ICC (ICC > 0.6). For all methods, the highest scores were obtained in the prefrontal GM region and the temporal and occipital GM/WM regions. The lowest ICC scores were reported in the frontal GM and WM regions. Overall, ICC scores from raw data were slightly higher than those from the denoised data.

## 4. Discussion

In this study, we have evaluated the reproducibility of the intra- and extra-cellular *T*_2_ relaxation time estimated by three non-parametric reconstruction techniques using a unique multi-echo *T*_2_ MRI dataset acquired in different subjects, scanning sites, and in separate sessions and runs. Moreover, two scenarios were tested with respect to the T2 estimation: first, estimating from the raw data (without any processing) and second, estimating from the data after denoising. An important contribution of our study is the analysis of reproducibility and stability in both GM and WM tissue types.

As the $T_{2}^{IE}$ is less affected by partial volume effects (PVE) than the voxelwise mean *T*_2_ estimated by other standard quantitative MRI techniques, we hypothesized that it could be a helpful imaging biomarker and an alternative or complement to the myelin water fraction. PVE appear especially close to the brain cortex, where voxels may contain a mixture of tissue and free water (e.g., GM/CSF) due to the highly convoluted geometry of the cortical mantle. As a result, the voxelwise mean *T*_2_ will be the average of $T_{2}^{IE}$ and the *T*_2_ of free water, weighted by their relative volumes. Thus, the voxelwise mean *T*_2_ may depend on the voxel location. This issue does not affect the $T_{2}^{IE}$ estimated in this study since it is computed from the *T*_2_ spectrum by discarding the portion corresponding to the myelin water and free water. Consequently, the intra- and extra-cellular *T*_2_ relaxation time may provide more specific information about potential abnormalities within the intra- and extra-cellular tissue compartments.

Interestingly, our results point to relatively higher stability of the $T_{2}^{IE}$ in the GM compared to WM. Hence, our findings suggest that the $T_{2}^{IE}$ could be a relevant metric for studying pathological conditions in the GM. A plausible explanation for this finding might be related to the complexity of the distribution of *T*_2_ times in these brain regions. While the *T*_2_ spectrum in GM commonly has a single lobe (or two very well separated lobes, when a portion of the voxel contains free water), in WM, there is a dominant lobe encoding the information from the intra- and extra-axonal water and a non-dominant lobe representing the myelin water. Estimating the myelin water lobe is difficult because (1) the myelin water signal decays very fast, and therefore, few data points contain relevant information about this water pool, and (2) the relative volume fraction of the myelin water is smaller than that of the intra- and extra-axonal water. Hence, its quantification is more affected by the underlying noise. Any error in estimating the right location and amplitude of the myelin water could increase the uncertainty in assessing the dominant lobe, i.e., $T_{2}^{IE}$ in the WM. In contrast, the $T_{2}^{IE}$ in GM is less affected by this issue. Moreover, the exchange between the intra- and extra-cellular water in GM is more significant, which tends to homogenize the spectrum, further reducing its complexity/variance.

### 4.1. Estimated variability in comparison to previous studies

Two primary metrics were employed to characterize the performance of the evaluated methods, the coefficient of variation (CoV) and the intra-class correlation (ICC). The range of values for the CoV resulting from the evaluated methods (i.e., in the range 0.32 and 1.64%) are smaller than those obtained in similar neuroimaging studies using other scalar metrics obtained from diffusion MRI data (in WM), and are concordant with previous studies using multi-echo *T*_2_ relaxometry data. For example, the median CoV values reported in the MASiVar study (21) for the fractional anisotropy and mean diffusivity varied from 3.34 to 11.95%, and from 1.37 to 5.12%, respectively (21). On the other hand, an average within-subject coefficient of variation (CoV) of 5.9% for the MWF metric was reported in Drenthen et al. (20). In contrast, Lee et al. (19) reported a mean inter-site MWF CoV across participants of 2.77% in the global WM. The only two previous studies that evaluated the $T_{2}^{IE}$ metric found a mean longitudinal CoV of 4% using 1.5T data and ROIs in WM and subcortical structures (11) and intra-site and inter-site CoVs of 0.51 and 0.31% in WM (17), respectively.

It is important to note that although other previous studies assessed the reproducibility of the parameters estimated from multi-echo *T*_2_ data, they mainly focused on characterizing the myelin water fraction (11, 16–20). The only two studies that analyzed the $T_{2}^{IE}$ time (11, 20) exclusively considered WM regions and subcortical structures. Therefore, to the best of our knowledge, this is the first study evaluating the reproducibility of $T_{2}^{IE}$ in the cortical GM.

### 4.2. Local microstructure sensitivity

The whole-brain voxelwise mean and standard deviation maps displayed in Figure 3 show a rich anatomical contrast provided by the $T_{2}^{IE}$. It suggests that this parameter is sensitive to the local microstructure and chemical properties. Notably, higher $T_{2}^{IE}$ values are located in GM and WM tracks with potentially higher axon calibers (e.g., the corticospinal tract and the optic radiations) (41), and smaller values are found in subcortical structures (e.g., putamen, pallidum) which may be affected by iron deposition, which is well-know to alter the magnetic field homogeneity and to decrease the *T*_2_. Mean $T_{2}^{IE}$ maps with similar image contrasts were previously reported in a multi-parametric atlas throughout the adult life span (42).

### 4.3. Estimation methods for $T_{2}^{IE}$

Results from the ICC metric indicate that the regularized NNLS method based on the L-curve−*I* is the best method for estimating $T_{2}^{IE}$, in terms of reproducibility. Interestingly, a previous study reported a superior performance of the MIML algorithm to estimate the MWF in WM Yu et al. However, as the performance of both methods for estimating $T_{2}^{IE}$ was not compared, it is not possible to know if our findings are agreement or disagreement with those reported in Yu et al. (25). We noticed that a potential bias in the evaluation could emerge as the MIML algorithm was trained for noisy data and, as such, results from denoised data may be sup-optimal. Nevertheless, we can discard this issue because our evaluation was conducted for both raw and denoised data, and results from both datasets showed a similar pattern. In fact, results from raw data were slightly more reproducible than those from denoised data, at a ROI level. Regarding the variability in different brain regions, the lowest ICC was found in frontal lobe regions and the highest in parietal regions, where the ICC strongly depended on the reconstruction method, being the L-curve−*I* the one that performed the best, and MIML the worst. The lower ICC found in frontal regions could be explained by a higher level of local noise, MRI-related artifacts, and residual errors in estimating the flip angle due to magnetic field inhomogeneity.

### 4.4. Limitations

This study has certain limitations. First, all the analyses are based on the same multi-echo *T*_2_ MRI sequence. Future studies should compare additional acquisition parameters and data collected from different MRI vendors (e.g., Siemens, Philips, GE). Second, all the participants were young healthy subjects. While this allowed us to reduce age-related differences, additional analyses comparing the sensitivity of the employed reconstruction methods to detect age-related differences were not possible. Third, future studies could evaluate the sensitivity of $T_{2}^{IE}$ to detect abnormalities in patients, as well as the comparison of the different metrics derived from multi-component *T*_2_ analyzes [see (43)]. Fourth, while the implemented EPG framework allowed us to study the variation in the MRI signals as a function of flip angle error, other factors including magnetization transfer effects and exchange (44), and the effect of diffusion due to internal gradients (45) caused by magnetic susceptibility differences at the fluid-tissue interfaces, were not considered. However, this is a standard assumption in multi-echo *T*_2_ relaxometry and its generalization would imply the development and validation of new models, which is beyond the scope of this work. Five, despite the fact that $T_{2}^{IE}$ is relatively immune to partial volume effects, we noticed image gradients around the boundary between GM and CSF. Nevertheless, as these gradients were not present in the individual images, we conclude that they were introduced by the normalization process, e.g., due to minor registration inconsistencies at the group level, and the linear interpolation (i.e., smoothing) done by the registration algorithm at the subject level. Finally, although we evaluated three reconstruction methods, additional algorithms could be analyzed, including the recently proposed Bayesian NNLS method (14) and the spatially regularized technique proposed by Kumar et al. (46).

## 5. Conclusion

We have acquired a unique multi-echo T2 MRI dataset to characterize the variability and reproducibility of the intra- and extra-cellular T2 relaxation time ($T_{2}^{IE}$). Moreover, we compared the estimates from three different reconstruction methods, including two classical algorithms based on regularized non-negative least squares and a novel machine learning approach trained with synthetic data. The analysis was conducted by using raw and denoised data, separately. We found that the smallest source of variance is the run (i.e., inter-run), followed by inter-session, inter-scanner, and inter-subject effects, respectively. Notably, there were no statistical differences between the inter-session and inter-scanner effects for any of the evaluated reconstruction techniques, suggesting that the acquisition sequence and employed methodology may be used in multi-site neuroimaging studies. Results from raw data were slightly more reproducible than those from denoised data. To the best of our knowledge, this is the first work reporting the variability and reproducibility of $T_{2}^{IE}$ across the cortical mantle, globally and in different brain lobes. Interestingly, the variability in the GM was smaller than that in the WM. Therefore, $T_{2}^{IE}$ could be a helpful imaging biomarker to characterize microstructure and molecular abnormalities in a range of pathological conditions in both GM and WM tissue types. Finally, the non-negative least squares method based on the L-curve technique produced the lowest intra-class correlation; therefore, it may be the preferred method for estimating the $T_{2}^{IE}$ time.

## Data availability statement

The datasets presented in this article are not readily available because requires a formal data sharing agreement. Requests to access the datasets should be directed to friedhelm.hummel@epfl.ch.

## Ethics statement

The studies involving human participants were reviewed and approved by Vaud Cantonal Ethics Committee (Switzerland) Project number: 2018-01355. The patients/participants provided their written informed consent to participate in this study.

## Author contributions

EF-G, GG, PK, MP, JB, GP, TH, AD, TK, EC-R, FH, and J-PT formulated the research goals and aims. EF-G, GG, MP, GP, TH, TY, SS, AD, TK, and EC-R design the methodology and analyzed the data. PK, JB, AC-M, EB, C-HP, TM, and MW collected the data. EF-G, GG, and EC-R wrote the original draft. EF-G, GG, PK, MP, GP, TH, TY, TK, EC-R, and FH reviewed and edited the manuscript. FH and J-PT acquired the financial support to perform the study. All authors contributed to the article and approved the submitted version.

## Funding

MP acknowledges the European Union's Horizon 2020 research and innovation program under the Marie Skłodowska-Curie grant agreement No 754462. EC-R was supported by the Swiss National Science Foundation (Ambizione grant PZ00P2-185814). This study was supported by Personalized Health and Related Technologies grant (PHRT-2017-205) of the ETH Domain (to FH) and by the Defitech Foundation (to FH). Open access funding provided by École Polytechnique Fédérale de Lausanne.

## Conflict of interest

Authors GP, TH, TY, and TK are employees of Siemens Healthineers International AG, Switzerland. The remaining authors declare that the research was conducted in the absence of any commercial or financial relationships that could be construed as a potential conflict of interest.

## Publisher's note

All claims expressed in this article are solely those of the authors and do not necessarily represent those of their affiliated organizations, or those of the publisher, the editors and the reviewers. Any product that may be evaluated in this article, or claim that may be made by its manufacturer, is not guaranteed or endorsed by the publisher.
